# CINV1 and CINV2 are required for increased tolerance to diverse stresses after ethylene-pretreatment of germinating seeds

**DOI:** 10.1371/journal.pone.0328236

**Published:** 2025-07-15

**Authors:** Esha Dutta, Mansi Patel, Colton Goodman, Allison L. Smith, Daniel M. Roberts, Brad M. Binder

**Affiliations:** 1 Genome Science and Technology Program, University of Tennessee Knoxville, Knoxville, Tennessee, United States of America; 2 Department of Biochemistry & Cellular and Molecular Biology, University of Tennessee Knoxville, Knoxville, Tennessee, United States of America; Hainan University, CHINA

## Abstract

Increasing plant vigor is a major challenge because land plants are vulnerable to many stresses which impacts their survival and reduces crop production. Here, we demonstrate that pretreatment of germinating *Arabidopsis thaliana* seeds with ethylene in darkness followed by transition to light leads to increased stress tolerance to a variety of stresses including high heat, high salt, heavy metal, re-oxygenation after hypoxia, cold, and flagellin 22 peptide. Under the conditions used, survival without ethylene pretreatment ranged from approximately 5% to 50% depending on the stressor. After ethylene pretreatment, survival ranged from 80% to 100% under the conditions studied. Only one stress examined, freezing, was unaffected by ethylene pretreatment. Ethylene pretreatment led to increases in sucrose, fructose, and glucose prior to stress; the levels of glucose after heat stress remained high. Additionally, ethylene pretreatment prevented reactive oxygen species (ROS) accumulation in leaves after high heat stress. Two cytosolic invertases, CINV1 and CINV2, were required for ethylene-mediated increases in survival and sugar levels. In contrast to wild-type seedlings, ethylene pretreatment did not prevent ROS accumulation after high heat stress in *cinv1;cinv2* double mutants. This suggests that normal breakdown of sucrose is required for these responses after pretreatment with ethylene.

## Introduction

Land plants are vulnerable to many stresses that impact survival. Additionally, global climate changes are leading to more extreme and unpredictable weather events leading to more stress such as flooding, increased salinity, and more extremes in temperature [1–3]. Stress often results in reductions in growth and crop yields [4–6]. To adapt to stresses, plants use a variety of mechanisms such as regulation of stress tolerance genes, synthesis of secondary metabolites, changes in metabolism, and upregulation of compounds to scavenge reactive oxygen species (ROS) [7–9]. These responses to stresses are often coordinated by one or more hormones [10–12].

Transient treatment with specific effector molecules, including hormones, can lead to higher stress tolerance, a process termed “chemical priming” [13–16]. Ethylene is a plant hormone that impacts growth, development, and responses to various environmental stresses [17]. Among its many effects, ethylene stimulates fruit ripening, leaf and petal abscission, and senescence, which can lead to reduced shelf life of fruits, vegetables, and cut flowers resulting in monetary losses. Transient treatment of plants or seeds with ethylene, the ethylene precursor 1-aminocyclopropane-1-carboxylic acid (ACC) to increase ethylene biosynthesis, or the ethylene-releasing chemical ethephon (2-chloroethylphosphonic acid) primes plants to have increased tolerance to high salt [18–20], high temperature [21,22], drought [23], and hypoxia, flooding, and re-oxygenation after hypoxia [24–28]. The effects of ethylene-mediated priming also extend to fruits since transient treatment of tomato fruits with ethephon reduced damage from cold [29]. However, the timing and dosage of ethylene pretreatment is critical and can also result in lower stress tolerance [30].

We recently reported that priming by transiently treating germinating seeds in darkness with ethylene increased growth and stress tolerance to salt stress, heat stress, and re-oxygenation stress after hypoxia [31]. The ethylene pretreated seedlings also exhibited increased carbon fixation and large increases in the levels of starch, sucrose, and glucose. Non-targeted metabolomics and transcriptomics revealed increases in many processes related to cell division, photosynthesis, and carbohydrate and amino acid metabolism. Two cytosolic invertases, CINV1 and CINV2 play important roles in plant growth [32,33]. The increase in growth from ethylene pretreatment required CINV1 and CINV2 and ethylene pretreatment led to increased transcript levels of both *CINV1* and *CINV2* pointing to breakdown of sucrose as a critical component necessary for increased growth after treatment with ethylene [31].

We wished to know whether the increased stress tolerance was limited to the stresses studied or was more general to many stresses. Therefore, we further characterized the effects of ethylene pretreatment of germinating seeds on more stresses and examined the effects of *cinv1;cinv2* double mutants on stress tolerance and soluble sugar levels. Our data indicate that ethylene pretreatment increases tolerance to many, but not all, stresses. Ethylene pretreatment led to increases in sucrose, fructose, and glucose and glucose levels remained high shortly after high heat stress. Moreover, transient treatment with ethylene resulted in lower levels of ROS in leaves after heat stress. CINV1 and CINV2, were required for these effects of ethylene suggesting that these responses require normal breakdown of sucrose. These results expand upon what we know about pretreatment with ethylene under these conditions by showing the importance of soluble sugars and sugar metabolism by CINV1 and CINV2 to increase stress tolerance.

## Results

### Ethylene pretreatment enhances tolerance to multiple stresses

We previously determined that transiently treating germinating seeds in darkness followed by removal of ethylene and transitioning to light led to plants that grew bigger and had higher tolerance to high temperature, high salt, and re-oxygenation after hypoxia [31]. We wished to know whether the increased stress tolerance was limited to these stresses or was more general. Therefore, we tested the stress tolerance of ethylene pretreated and untreated seedlings to these same stresses as well as stress to high copper, cold, freezing, and flagellin22 (flg22) peptide. Flg22 peptide elicits pattern triggered immunity and was chosen to mimic bacterial pathogens [34]. Additionally, published protocols for some stresses such as high heat stress [35,36] and re-oxygenation after hypoxia [37] are carried out in darkness. And, cold and freezing stress experiments were conducted in darkness. Given the importance of carbohydrate metabolism in increased growth after ethylene pretreatment [31], we also explored whether darkness was an additional factor in enhanced stress tolerance after treatment with ethylene by also examining heat stress in light. For these assays we germinated the seeds in darkness for three days (seeds on agar) or four days (seeds in soil) in the presence (ethylene pretreated) or absence (no ethylene) of 0.7 ppm ethylene. Ethylene was then removed and the seedlings transferred to light (16 h photoperiod) for either five or seven days (as designated) before stress treatment. The seedlings were then assessed for survival at the indicated times after stress (Summarized in Table 1).

**Table 1:** Summary of stress conditions used to determine stress tolerance.

Stress	Summary of treatment protocol	Time after stress for survival quantification
High heat in darkness	five days after ethylene pretreatment exposed to 45 °C for 30 min in darkness	3 days
High heat in light	five days after ethylene pretreatment exposed to 45 °C for 30 min in light	1 day
Re-oxygenation after hypoxia in darkness	five days after ethylene pretreatment exposed to < 2% O_2_ for 6 hours followed by return to normoxic conditions	3 days
High Salt in light	five days after ethylene pretreatment transferred to plates with 150 mM NaCl	3 days
Heavy metal in light	five days after ethylene pretreatment transferred to plates with 50 µM CuSO_4_	3 days
Cold in darkness	seven days after ethylene pretreatment exposed to 0 °C for 24 hours	7 days
Freezing in darkness	seven days after ethylene pretreatment exposed to −20 °C for 2 hours	2 days
flg22 in light	seven days after ethylene pretreatment transferred to plates with 1 µM flg22	7 days

**Fig 1:**
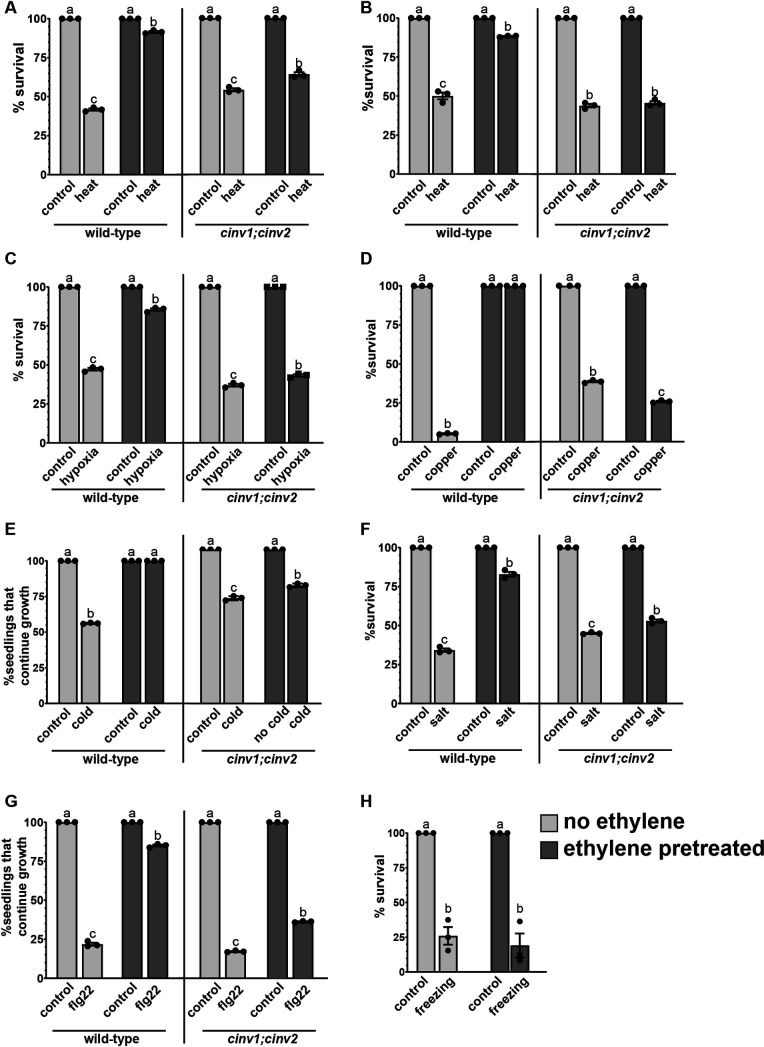
Ethylene pretreatment increases survival to multiple stresses. Germinating wild-type and *cinv1;cinv2* seeds were exposed to 0.7 ppm ethylene (ethylene pretreated) or ethylene-free air (no ethylene) in darkness for three days. At this time, they were transferred to ethylene-free conditions and grown under a 16 h photoperiod for the indicated times prior to being treated with the indicated stress or kept in control conditions. After all stresses, plants were returned to light under a 16 h photoperiod. A) Five days after ethylene pretreatment, seedlings were stressed with 45 °C in darkness for 30 minutes and then returned to room temperature and grown under light. Control seedlings were placed in darkness at 22 °C for 30 minutes. Survival was assessed three days later. B) Five days after ethylene pretreatment, seedlings were stressed with 45 °C in light for 30 minutes and then returned to room temperature and grown under light. Control seedlings were kept at 22 °C. Survival was assessed one day later. C) Five days after ethylene pretreatment, seedlings were exposed to hypoxia conditions in darkness for 6 hours, then returned to normoxic conditions and grown under light. Control seedlings were kept in darkness for 6 hours in normoxic conditions. Survival was measured three days later. D) Five days after ethylene pretreatment, seedlings were transferred to plates containing 50 µM CuSO_4_ or no added CuSO_4_ (control). Survival was quantified seven days later. E) Seven days after ethylene pretreatment, seedlings were transferred to an ice bath for one day in darkness. Control seedlings were kept at room temperature in darkness. Both were then grown in light for seven days before survival was measured. F) Five days after ethylene pretreatment, seedlings were transferred to plates containing 150 mM NaCl or no added NaCl (control). Survival was determined three days later. G) Seven days after ethylene pretreatment, seedlings were transferred to plates containing 1µM flg22 or no added flg22 (control). Survival was assessed seven days later. H) Seven days after ethylene pretreatment, wild-type seedlings were transferred into a freezer (−20 °C) for two hours in darkness. Control seedlings were kept at room temperature in darkness. Both were then grown in light for two days before survival was measured. Data is the average ± SEM of at least 30 seedlings. Different letters denote statistical difference (*P *< 0.05) using ANOVA.

In all conditions, unstressed wild-type *A. thaliana* seedlings had 100% survival whether or not pretreated with ethylene (Fig 1). Seedlings not pretreated with ethylene and exposed to heat stress (45 °C) in darkness, heat stress in light, re-oxygenation after 6 hours of hypoxia stress, high copper (50 µM CuSO_4_), or high salt (150 mM NaCl) had poor survival ranging from approximately 5% (copper) to 25−40% (re-oxygenation after hypoxia, high heat in darkness or light, high salt, freezing). Transiently treating germinating seeds in darkness with ethylene followed by removal of ethylene and growth under light led to statistically significant increases in survival after these stresses. Cold stress (0 °C for 24 hours) and application of 1 µM flg22 to seedlings not pretreated with ethylene led to a different response where all seedlings stayed green, but many stopped growing in the time-frame of the experiments. Ethylene pretreated seedlings exposed to these two stresses had statistically more seedlings that continued to grow. Only one stress out of those tested was unaffected by ethylene pretreatment. Exposure to freezing stress (2 hours at −20 °C) led to about 75% death for both ethylene pretreated and untreated seedlings, showing that ethylene pretreatment does not affect survival to this stress. These data indicate that survival and growth in response to many, but not all, stresses are increased by ethylene pretreatment under the conditions tested.

**Fig 2:**
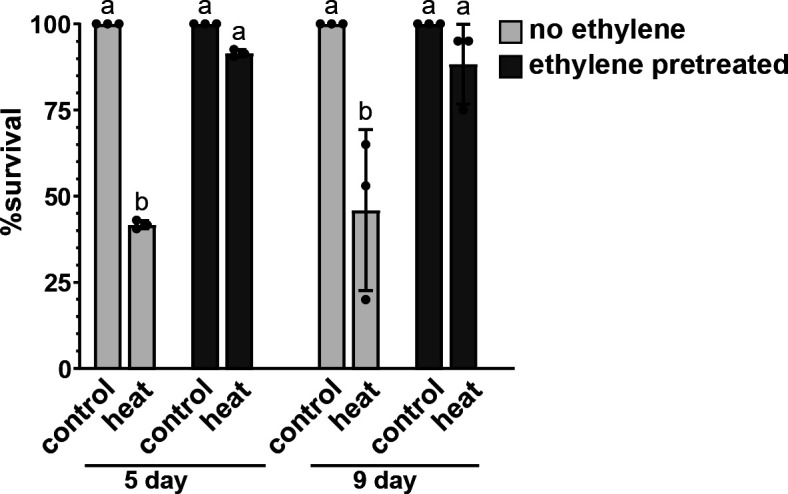
Enhanced stress tolerance is not due to accelerated development. Germinating wild-type seeds were exposed to 0.7 ppm ethylene (ethylene pretreated) or ethylene-free air (no ethylene) in darkness for three days. At this time, they were transferred to ethylene-free conditions and grown under a 16 h photoperiod for either five or nine days prior to being treated with 45 °C in darkness for 30 minutes and then returned to room temperature and grown under light. Control seedlings were at 22 °C. Survival was assessed three days later. Data is the average ± SEM of at least 30 seedlings. Different letters denote statistical difference (*P *< 0.05) using ANOVA.

One explanation for increased stress tolerance is simply that the ethylene pretreated seedlings are growing faster and thus are more stress tolerant because they are more advanced in development. If this is true, we predicted that older seedlings not pretreated with ethylene would have better survival compared to younger control seedlings, and this better survival would be comparable to younger seedlings that had been pretreated with ethylene. Our prior research showed that nine days after transfer to light, seedlings not pretreated with ethylene have primary roots approximately the same length as ethylene pretreated seedlings five days after removal of ethylene transfer and to light [31]. Therefore, to test whether developmental differences of seedlings affected stress tolerance, we compared heat stress tolerance in darkness in seedlings five days and nine days after removal of ethylene and transfer to light. Survival of seedlings not pretreated with ethylene was similar at both time-points (Fig 2). Additionally, the survival of stressed seedlings not pretreated with ethylene nine days after transfer to light was lower than ethylene pretreated stressed seedlings five days after transfer to light. Thus, these data do not support the model that increased stress tolerance is due to more accelerated development after ethylene pretreatment.

### CINV1 and CINV2 are required for ethylene-mediated enhanced stress tolerance to multiple stresses

The cytosolic invertases CINV1 and CINV2 are required for increased survival to heat stress in darkness [31]. Darkness can affect sugar levels and these invertases break down sucrose to glucose and fructose. This led us to question whether or not CINV1 and CINV2 only affected survival after stress in darkness, or if this was a more general requirement for enhanced survival after ethylene pretreatment. To resolve these possible interpretations, we examined ethylene pretreated and untreated *cinv1;cinv2* seedlings in response to heat stress in darkness and heat stress in light. We also evaluated whether CINV1 and CINV2 are required for enhanced survival after other stresses leading us to examine the ability of *cinv1;cinv2* seedlings to survive re-oxygenation after hypoxia stress in darkness, high salt, high copper, cold stress in darkness, and flg22. Like wild-type seedlings, unstressed *cinv1;cinv2* had 100% survival in both ethylene untreated and ethylene pretreated conditions (Fig 1). Ethylene untreated *cinv1;cinv2* seedlings exposed to stress showed similar responses to wild-type seedlings, although, *cinv1;cinv2* seedlings had better survival than wild-type in response to copper stress. Ethylene pretreatment did not significantly improve survival or growth of *cinv1;cinv2* seedlings after any of the stresses tested. Thus, these two invertases are required for increased tolerance to these stresses.

### Exogenous sucrose affects responses to ethylene pretreatment

In wild-type seedlings, exogenous sucrose increases growth and masks the effect of ethylene pretreatment on growth. Additionally, exogenous sucrose increases survival in response to heat stress in darkness in seedlings not pretreated with ethylene, but results in worse survival of seedlings pretreated with ethylene than seedlings grown in the absence of sucrose [31]. To determine whether this applied to other stresses, we examined survival during re-oxygenation after hypoxia.

**Fig 3:**
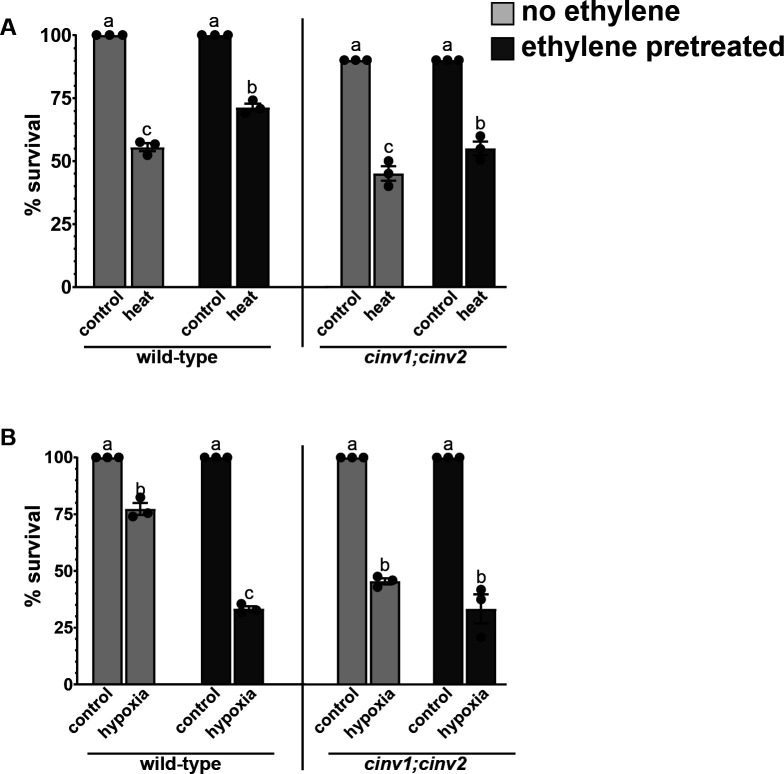
Exogenous sucrose affects survival after ethylene transient exposure to ethylene. Germinating wild-type and *cinv1;cinv2* seeds grown in the presence of 0.2% (w/v) sucrose were exposed to 0.7 ppm ethylene (ethylene pretreated) or ethylene-free air (no ethylene) in darkness for three days. At this time, they were transferred to ethylene-free conditions and grown under a 16 h photoperiod for five days. A) Five days after ethylene pretreatment, seedlings were stressed with 45 °C in darkness for 30 minutes and then returned to room temperature and grown under light. Control seedlings were at 22 °C Survival was assessed three days later. B) Five days after ethylene pretreatment, seedlings were exposed to hypoxia conditions in darkness for 6 hours, then returned to normoxic conditions and grown under light. Control seedlings were kept in darkness for 6 hours in normoxic conditions. Survival was measured three days later. Data is the average ± SEM of at least 30 seedlings. Different letters denote statistical difference (*P *< 0.05) using ANOVA.

All non-stressed seedlings had 100% survival (Fig 3). As previously reported [31], ethylene untreated wild-type seedlings grown on 0.2% (w/v) sucrose had slightly higher survival to high heat in darkness than seedlings grown in the absence of sucrose (Figs 1, 3). Also, in the presence of sucrose the enhancement of survival from ethylene pretreatment was reduced slightly compared to seedlings pretreated with ethylene in the absence of exogenous sucrose (Figs 1, 3).

Exogenous sucrose also increased survival of ethylene untreated seedlings during re-oxygenation after hypoxia. However, in contrast to high heat in darkness, the addition of exogenous sucrose led to very low survival of ethylene pretreated wild-type seedlings below the survival of seedlings not pretreated with ethylene in the absence of sucrose (Figs 1, 3). This indicates that under certain conditions exogenous sucrose can block the effects of ethylene pretreatment. Addition of sucrose to *cinv1;cinv2* seedlings had no major effect on survival to either stress in ethylene-pretreated or untreated conditions compared to the absence of added sucrose. These data further support the idea that CINV1 and CINV2 are important for enhanced stress tolerance in response to transient exposure to ethylene.

### The levels of glucose and fructose increase in response to pretreatment with ethylene

**Fig 4:**
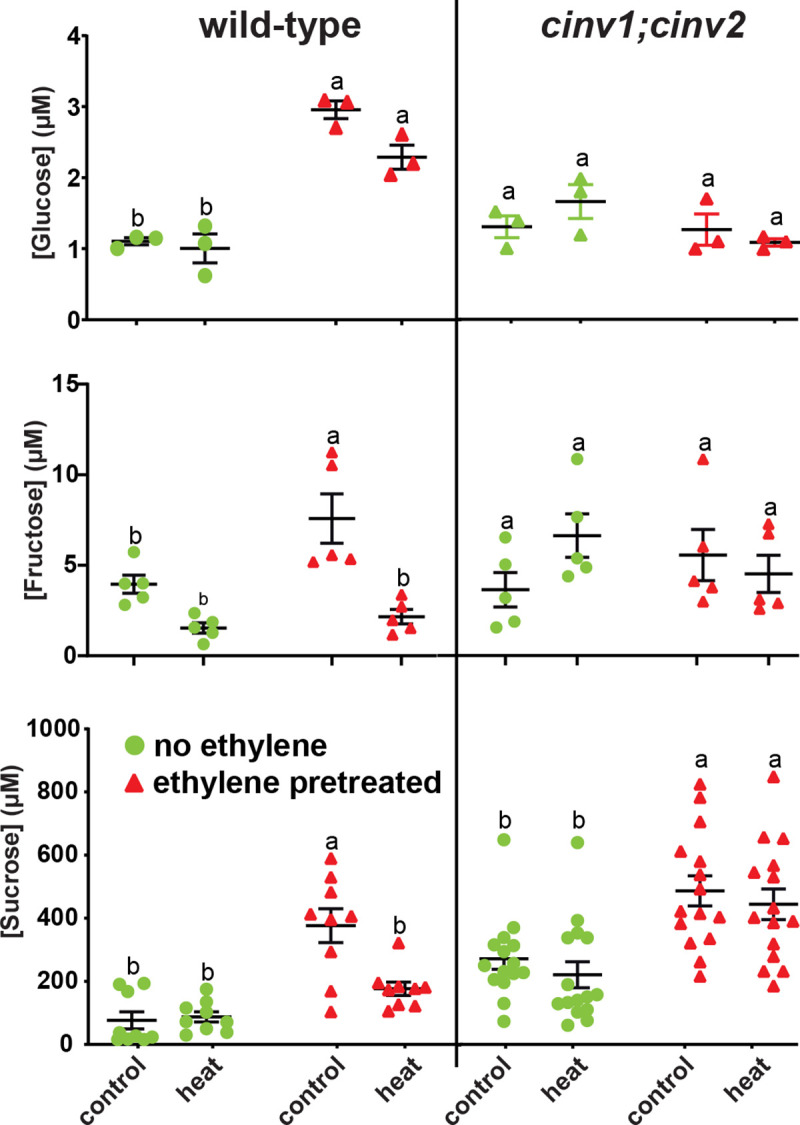
Ethylene pretreatment affects soluble sugar accumulation after stress. Germinating wild-type and *cinv1;cinv2* seeds were exposed to 0.7 ppm ethylene (ethylene pretreated) or ethylene-free air (no ethylene) in darkness for three days. At this time, they were transferred to ethylene-free conditions and grown under a 16 h photoperiod for five days and then were exposed to either A) high temperature (45 °C) in darkness or B) hypoxia in darkness for 6 hours, then returned to normoxic conditions. Unstressed seedlings as described in Fig 1 were used as controls. One hour after the stress, the levels of sucrose, glucose, and fructose were quantified. The mean ± SEM is plotted. Different letters denote statistical difference (*P* < 0.05) using ANOVA.

Previously we showed that sucrose and glucose levels are higher in seedlings several days after transient treatment with ethylene [31]. Responses to stress can be rapid. Therefore, we examined for more rapid changes by sampling one hour after stress. In wild-type seedlings not pretreated with ethylene, the levels of these sugars was unchanged by heat stress in darkness (Fig 4). In unstressed seedlings, ethylene pretreatment led to higher levels of sucrose, glucose, and fructose compared to controls not pretreated with ethylene. After heat stress in darkness, the levels of glucose remained high in ethylene pretreated seedlings. By contrast, fructose and sucrose levels were lower and were comparable to levels observed in the seedlings not pretreated with ethylene indicating that they are rapidly used after this stress.

Since CINV1 and CINV2 are important for the breakdown of sucrose to glucose and fructose [32,33], we measured sugar levels in *cinv1;cinv2* seedlings. In contrast to wild-type seedlings, ethylene pretreatment had no measurable effect on the levels of glucose or fructose in the *cinv1;cinv2* mutants. Additionally, heat stress did not alter the levels of either monosaccharide in either ethylene pretreated or untreated plants. Sucrose levels were raised by ethylene pretreatment and in contrast to wild-type seedlings, sucrose levels remained high in ethylene pretreatment, heat-stressed *cinv1;cinv2* seedlings. Thus, normal accumulation of these sugars in response to ethylene pretreatment and heat stress requires CINV1 and CINV2.

### Ethylene pretreatment reduces ROS levels

Many stresses lead to an increase in ROS which can lead to cellular damage. We therefore wished to know whether or not pretreatment with ethylene affected ROS levels after stress. To examine this we compared the ROS levels in ethylene pretreated and untreated wild-type seedlings one hour after exposure to heat stress in darkness. For comparison, we also examined seedlings maintained at non-stressful temperature.

**Fig 5:**
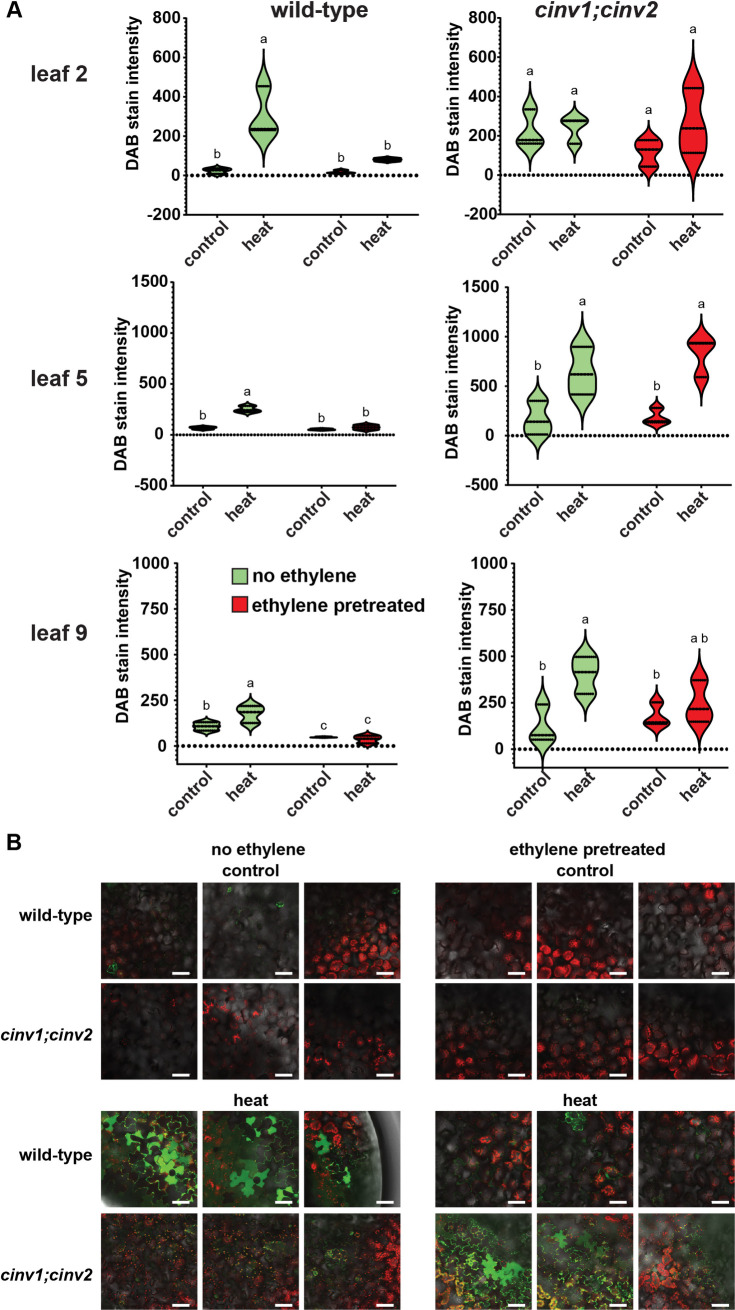
Ethylene pretreatment affects ROS accumulation after heat stress in darkness. Germinating wild-type and *cinv1;cinv2* seeds were exposed to 0.7 ppm ethylene (ethylene pretreated) or ethylene-free air (no ethylene) in darkness for three days. At this time, they were transferred to ethylene-free conditions and grown under a 16 h photoperiod for five days. They were then exposed to either high temperature (45 °C) or control conditions (22 °C) in darkness for 30 minutes. A) One hour later the samples were stained with DAB to visualize H_2_O_2_ and images acquired. The amount of DAB staining in leaves three, five and nine was quantified and the mean ± SEM is plotted. Different letters denote statistical difference (*P* < 0.05) using ANOVA. B) At 45 minutes after stress, DCF fluorescence in leaf cells was imaged with confocal microscopy. Select merged images of cells from three leaves are shown for each condition. Images show merged channels for chlorophyll autofluorescence (red), DCF fluorescence (green), and DIC. Scale bars = 50 µm.

We first determined the levels H_2_O_2_ using 3,3’-diaminobenzidine (DAB) staining (Fig 5a, S1). For this we compared DAB staining in leaves two, five, and nine. All three leaves showed similar patterns of DAB staining. Heat stress led to an increase in DAB staining in wild-type seedlings not pretreated with ethylene. Ethylene pretreatment led to a reduction of DAB staining in leaf nine of non-stressed seedlings and eliminated any statistically significant increase in DAB staining levels after heat stress for all three aged leaves.

We also used dichlorofluorescein (DCF) to fluorescently visualize ROS in wild-type leaf cells (Fig 5b). Unstressed wild-type seedlings had little or no DCF fluorescence whether ethylene pretreated or not. After heat stress, the wild-type seedlings not pretreated with ethylene had high levels of DCF fluorescence that filled many cells suggestive of wide-spread cellular damage. By contrast, ethylene pretreated, heat-stressed seedlings had less DCF fluorescence, and, where present was restricted to the cell periphery.

Together, these data indicate the ethylene pretreatment results in less ROS accumulation in wild-type leaves shortly after heat stress in darkness. Additionally, pretreatment may reduce cellular damage even when ROS accumulates.

### CINV1 and CINV2 are required for ethylene-mediated reductions in ROS levels

Since our results above indicate that CINV1 and CINV2 are required for increased stress tolerance after pretreatment with ethylene, we examined ROS levels in *cinv1;cinv2* mutants. DAB staining revealed that *cinv1;cinv2* mutants generally have higher levels of H_2_O_2_ in the leaves of unstressed seedlings (Fig 5a). In leaf two, heat stress did not increase DAB staining levels and ethylene pretreatment had no measurable effect on DAB staining levels. By contrast, DAB staining increased in leaves five and nine after heat stress. Pretreatment with ethylene had no effect on this increase suggesting that modulation of H_2_O_2_ by ethylene pretreatment requires CINV1 and CINV2. DCF fluorescence revealed low levels of ROS in *cinv1;cinv2* seedlings that had not been stressed (Fig 5b). Heat stress led to an increase in DCF fluorescence in both ethylene pretreated and untreated *cinv1;cinv2* seedlings. DCF fluorescence and chlorophyl autofluorescence in untreated *cinv1;cinv2* seedlings co-localized in small, oval puncta (Figs 5b, S2). These are smaller than chloroplasts. Ethylene pretreated *cinv1;cinv2* showed significantly higher ROS levels than *cinv1;cinv2* not pretreated with ethylene and widespread cytosolic fluorescence from ROS accumulation indicating cellular damage.

## Discussion

Enhancing plant vigor is a challenge to feed a growing human population. This challenge is exacerbated by global climate changes resulting in more stress to plants such as flooding, increased salinity, and more extremes in temperature [1–3]. In general, stresses result in a reduction in growth leading to decreases in crop yields [4–6]. One approach that can increase stress tolerance is to treat plants with effector molecules leading to priming [13–16]. Ethylene has been studied as a priming agent to improve stress tolerance where either seeds or plants are exposed to ethylene, ACC to increase ethylene biosynthesis, or the ethylene-releasing compound ethephon [18–28,30,31]. Most of these prior studies examined responses to only one stress. In this study we showed that transiently treating germinating *A. thaliana* seeds in darkness with ethylene for several days, followed by removal of ethylene and transfer to light, resulted in seedlings with higher stress tolerance to multiple stresses including higher tolerance to high heat, cold, salt, heavy metal, re-oxygenation after hypoxia, and flg22.

The only stress we studied that was unaffected by ethylene pretreatment was freezing stress. Ethylene’s function in cold stress responses is complex and varies across species, developmental stages, and environmental contexts where it sometimes functions as positive regulator and other times as a negative regulator [38–41]. Nonetheless, it is surprising that ethylene pretreatment does not affect freezing survival since increases in soluble sugar levels, such as we observed in this study, correlate with increased survival to freezing [42]. Additionally, ethylene negatively regulates freezing tolerance in *A. thaliana* by down-regulating type-A *ARR* genes (Shi et al., 2012). Our prior RNA-seq data indicate that several type-A *ARR* genes are up-regulated by pretreatment with ethylene [31] which should also increase survival to freezing. Freezing leads to the formation of ice crystals in plants that can cause physical damage to cells; increased levels of soluble sugars can reduce ice crystal formation [43–45]. It is possible that the effects of ethylene pretreatment on sugar levels were not large enough to prevent ice crystal formation. Another explanation for this may be that increased freezing tolerance has been linked to decreased photosynthesis [46], whereas, ethylene-mediated metabolic priming increases photosynthesis [31], perhaps counteracting the protective effects of increased soluble sugars. Also, our prior RNA-seq experiments [31] show that many genes encoding proteins related to responses to freezing stress, including several transcription factors, isoforms of 3-keto-coA-synthases, and cold regulated plasma membrane (COR) proteins, are down-regulated by ethylene pretreatment providing another possible mechanism underlying the poor survival of ethylene pretreated seedlings to freezing stress. Experiments with less drastic temperature shifts and gradual temperature declines from optimal to subzero levels might reveal more insights into freezing stress responses after ethylene pretreatment.

Previously, we showed that photosynthetically-derived sucrose and glucose levels are higher in ethylene pretreated plants compared to untreated plants [31]. Higher levels of sugars in response to stress have been linked to enhanced survival to various stresses [47,48]. Here we showed that wild-type seedlings pretreated with ethylene had higher levels of sucrose, fructose, and glucose when unstressed and maintained higher levels of glucose one hour after high heat stress. This correlates with the increased survival we observed for most stresses tested. Soluble sugars can also lead to higher ROS and contribute to cell death [49,50], which might explain why exogenous sucrose led to poor survival of ethylene pretreated seedlings during re-oxygenation after hypoxia. In contrast to wild-type, *cinv1;cinv2* mutants did not respond to ethylene pretreatment with increased survival to any of the stresses tested and failed to have an increase in glucose and fructose levels further supporting the idea that sugar accumulation is important for increased tolerance to stress. Whether this is due to more ROS buffering by the sugars themselves [48,50–53] or simply having more energy stores to survive a stress is an open question. Since *cinv1;cinv2* mutants also have altered carbon partitioning, leading to lower levels of sugar conjugates such as glucose 6-phosphate, fructose 6-phosphate, and UDP-glucose [32,33], it may also be that *cinv1;cinv2* plants failed to survive stress because of this altered partitioning.

Generation of ROS is a common outcome for both abiotic and biotic stresses in plants and there is a link between ethylene and ROS accumulation where application of ethylene triggers ROS accumulation and ethylene-insensitive mutants have a weaker oxidative burst in response to stress [54,55]. In this study, heat stress led to an increase in ROS levels in the leaves of seedlings not treated with ethylene. This increase was less in wild-type than *cinv1;cinv2* seedlings. Unlike wild-type seedlings, where ethylene pretreatment prevented ROS accumulation after heat stress, the increased accumulation of ROS in *cinv1;cinv2* seedlings after heat stress was largely unaffected by pretreatment with ethylene. This is likely responsible for the poor survival of these mutants even after transient exposure to ethylene. Thus, there is an inverse relationship between ROS levels and plant survival in response to heat stress. We hypothesize that ethylene pretreated plants have enhanced stress tolerance because less ROS accumulates resulting in less cellular damage.

Similar to our results pretreating germinating seeds with ethylene, others have noted that ethylene pretreatment of *A. thaliana* plants led to better survival to hypoxia and re-oxygenation after hypoxia that correlates with lower levels of ROS in the ethylene pretreated plants [26,28]. Similarly, treating rice seeds with ACC improved submergence stress tolerance [27] and ethephon treatment of drought sensitive wheat seeds led to better drought tolerance [23]. However, contrary to our results, the ethylene pretreated rice plants had higher ROS levels, and the ethylene pretreated wheat plants had reduced ROS scavenging capability and lower soluble sugar content. This suggests that increased stress tolerance from ethylene pretreatment may be different in rice and wheat compared to *A. thaliana*. Alternatively, the developmental stage at which ethylene is applied might affect the mechanisms by which stress tolerance is increased [30]. In addition to causing cellular damage, ROS can also function as a signal to help plants survive stress [5,56,57] which might be the underlying mechanism for the increased survival in rice and wheat. ROS signaling could also be a factor in our experiments, especially in response to elicitors like flg22 where rapid and transient ROS bursts are important for the innate immune response to pathogens [54]. Our prior RNA-seq data shows several genes encoding proteins involved in ROS signaling are altered by ethylene pretreatment [31] opening up the possibility that ethylene pretreatment is also altering ROS signal transduction to affect survival.

We propose a model in which pretreating with ethylene reduces cell damage by reducing ROS accumulation. However, the mechanism by which ROS levels are kept low after ethylene pretreatment is not known. In wild-type, glucose levels are high and ROS levels are low in ethylene pretreated, heat-stressed seedlings. By contrast, glucose levels remain low after ethylene pretreatment and ROS levels rise in *cinv1;cinv2* seedlings. Two general models can be considered from our data. One possibility is that soluble sugars such as glucose and sucrose are acting to scavenge ROS [48,50–53]. Alternatively, the increased levels of soluble sugars might promote survival because of higher levels of carbon as an energy source which allows the plant to devote resources to scavenging or enzymatically breaking down ROS. Support for both models is the inverse relationship between glucose compared to ROS in wild-type seedlings after heat stress.

Increased growth from ethylene pretreatment, at least in part, is due to higher levels of sugars contributing to metabolism [31], suggesting that during stress, this excess energy could be diverted to the management of ROS levels. Genes encoding proteins related to sugar and starch metabolism and distribution, such as SWEETs, starch synthases, and sucrose phosphate synthases, are increased by ethylene pretreatment [31]. Additionally, RNA-sequencing data shows an increase in transcript abundance for eight genes encoding glutathione S-transferase, which is a class of enzyme that breaks down ROS, and non-targeted metabolomics shows ethylene pretreatment leads to increases in several ROS scavengers, including glutamine, malate, and aspartate [31]. These models for the role of soluble sugars are not mutually exclusive, and both could be true. At this point, more research will be needed to test whether either of these models is correct.

These data show that pretreating germinating *A. thaliana* seeds in darkness can lead to increased stress tolerance to multiple stresses. We have previously observed growth enhancement in several plant species after ethylene pretreatment [31] suggesting that stress tolerance might also be increased in multiple species. If true, this ethylene pretreatment protocol represents a possible new approach to increase plant growth and stress tolerance in plants germinated in greenhouses and other enclosed conditions where plants can be easily pretreated with ethylene.

## Materials and methods

### Plant materials

All experiments used *Arabidopsis thaliana.* Wild-type (Columbia) seeds are lab stocks. The *cinv1;cinv2* seeds are in the Columbia background and were from Charles Anderson [32].

### Preparation of seeds and growth

*A. thaliana* seeds were prepared and grown on either 0.8% (w/v) agar containing half-strength Murashige and Skoog basal salt mixture, pH 6.0 [58] with no added sugar. In some experiments on agar, the medium was supplemented with the indicated concentration of other compounds. In some experiments, seeds were sown in soil as described by Brenya, Dutta (31).

### Pretreatment with ethylene

For seeds sown on agar, seeds were germinated in darkness in ethylene-free air or 700 ppb ethylene for 3 days. For seeds sown in soil, seeds were germinated in darkness in ethylene-free air or 700 ppb ethylene for 4 days. At this time, the seedlings were transferred to ethylene-free conditions and 155 μmol m^-2^ s^-1^ white light (16 hour photoperiod). At the indicated times after transfer out of ethylene and into light, plants were then exposed to various stresses as detailed below.

### Stress assays

Unless otherwise noted, stress assays were conducted on agar plates with each plate containing between 10 and 12 seedlings. There were three plates per condition and all experiments were repeated three times. Table 1 summarizes the stresses used.

### Heat tolerance assays

Heat tolerance was assessed as previously described [35,36] with slight modifications. Briefly, this was done by exposing seedlings at five days after transfer to light and removal of ethylene to either 22 °C (control) or 45 °C for 30 min in darkness followed by a return to 22 °C and white light as described previously. Any seedlings that turned white and had not grown new leaves by three days afterwards were scored as having died. In some experiments, heat stress was administered under the same conditions except the stress was administered under white light. Survival was determined one day after the stress.

### Hypoxia re-oxygenation assays

Hypoxia stress was applied by purging a sealable chamber (9.5 L Jar Gas Pak System, BBL) with argon gas to a final O_2_ level less than 2% measured by using a Traceable Oxygen probe (Fisher Scientific) as previously described [37]. Plants five days after ethylene pretreatment were kept in hypoxic conditions in darkness for 6 hours and then were returned to normal aerobic conditions and white light. Survival to re-oxygenation was then assessed three days after stress by monitoring for the absence of shoot chlorosis [59]. The normoxic (control) seedlings were treated identically except that argon purging was omitted.

### Salt stress assays

Five days after ethylene pretreatment, seeds were transferred to plates containing either 150 mM NaCl or no added NaCl. Survival was determined three days later. Any seedlings that turned white were scored as having died.

### Copper stress assays

Five days after ethylene pretreatment, seeds were transferred to plates containing either 50 µM CuSO_4_ or no added copper. Survival was determined seven days later. Any seedlings that turned white were scored as having died.

### Cold stress assays

Seven days after ethylene pretreatment, seedlings on agar plates were exposed to either control (22 °C) or cold (0 °C) by immersion in an ice bath in darkness for 24 hours. At this time the seedlings were returned to room temperature and grown in the light for seven days before survival was assessed.

### Freezing stress assays

Freezing stress was done on seeds sown in soil with 20 seeds per pot and three pots per condition. Seven days after ethylene pretreatment, the seedlings were exposed to freezing stress at −20 °C for 2 hours. At this time the seedlings were returned to room temperature (leaves of seedlings exposed to −20 °C were frozen hard) and grown in the light for 2 days before survival was assessed. Control seedlings were kept at room temperature (22 °C control) in darkness for 2 hours. This was scored by plants that regained turgidity [42].

### Flagellin assays

A 1 mM flg22 (PhytoTech Labs) stock solution was made by dissolving flg22 peptide in diethylpyrocarbonate-treated deionized water. Seeds were transferred seven days after ethylene pretreatment to plates containing either no flg22 (control) or 1 µM flg22. Survival was then measured assessed seven days after stress by monitoring for the absence of shoot chlorosis

### Sucrose, glucose, and fructose analyses

*A. thaliana s*eedlings were grown and treated with ethylene or ethylene-free air and exposed to either heat stress in darkness or hypoxia re-oxygenation stress as described above. One hour after the stress, samples were prepared for either sucrose, glucose, or fructose quantification. For all assays, seedlings were frozen in liquid nitrogen after being weighed and then ground into a fine powder in a 2 ml Corning screwcap Eppendorf tube containing 2–3 steel balls using a homogenizer (Biospec) for 15 seconds. Glucose quantification was done following the manufacturer’s instructions in the glucose assay kit (MAK476, Sigma-Aldrich). Fructose and Sucrose quantification were done using a modified protocol using reagents from the FA20 and SCA20 kits (Sigma-Aldrich). Briefly, all the assays were performed in 96 well plates on a microplate reader (Varioskan Lux Plate Reader). Each sample had at least three biological replicates and three technical replicates per condition and treatment for these assays.

### DAB staining to measure H_2_O_2_ levels

3,3′-Diaminobenzidine (DAB) (Sigma-Aldrich) staining using a protocol adapted from Daudi and O’Brien [60] was used on control and seedlings exposed to heat stress in darkness seven days after ethylene pretreatment. One hour after the stress, individual rosettes were cut off the seedlings and placed in a well of a 12-well plate. Staining solution consisting of 1 mg mL^-1^ DAB in a solution of Tween 20 (0.05% v/v) and 10 mM sodium phosphate buffer (pH 7.0) was added to the wells, followed by vacuum infiltration for 15 minutes. Staining was carried out overnight on a rotating shaker at room temperature in the dark. After staining, leaves from the rosettes were separated using forceps, and leaves two, five and nine were selected for imaging. To remove excess stain and clear chlorophyll, a bleaching solution containing ethanol: acetic acid: glycerol (3:1:1 v/v) was added to the leaves and heated to 95°C. The leaves were then viewed using an Olympus Vanox light microscope and images taken using a Canon EOS Rebel T1i camera. Quantification of staining was carried out using ImageJ (ver. 1.54p).

### Dichlorofluorescein (DCF) and confocal microscopy to image ROS

*A. thaliana* seedlings were grown on agar plates and treated with ethylene or ethylene-free air as detailed above. Five days after removal of ethylene and transfer to light, three seedlings per condition were taken and placed on a glass microscope slide and kept at either 25 °C or 45 °C for 30 min in the dark. This was followed by the addition of a general fluorescent ROS probe, 2’,7’-dichlorodihydrofluorescein diacetate (H2DCFDA, Invitrogen). This is cleaved in cells to release DCF which fluorescently labels ROS species [61–64]. To do this, a stock solution of 50 µM H2DCFDA was prepared by dissolving it in 0.1% (v/v) DMSO, from which a working solution of 5 µM H2DCFDA was used for incubating the seedlings in the dark for 15 minutes. Confocal images (Leica SP8) were taken using a 40X water immersion lens 45 minutes after stress. A white light laser was used for an excitation wavelength of 488 nm. DCF fluorescence emission was captured between 493–550 nm wavelengths. Chlorophyll autofluorescence emission was recorded between 650–750 nm. Images were processed using Leica Application Suite or LAS X software.

### Statistical analyses

Pairwise comparisons were conducted with Students *t*-tests using Excel (Office 365). For multiple group comparisons, ANOVA tests were performed using GraphPad Prism (ver. 10.4.l).

## Supporting information

S1 Fig Images of DAB-stained leaves. (PDF)

S2 Fig Close-up of DCF fluorescence in heat-stressed cinv1;cinv2 not pretreated with ethylene. (PDF)

S1 Data Underlying numerical data. (XLSX)
