# Supplementary material for: CINV1 and CINV2 are required for increased tolerance to diverse stresses after ethylene-pretreatment of germinating seeds
Source: PLoS One. 2025 Jul 15;20(7):e0328236. doi: 10.1371/journal.pone.0328236 (PMC12262900; doi:10.1371/journal.pone.0328236)
Supplement: S1 Fig — (PDF) [file pone.0328236.s001.pdf]

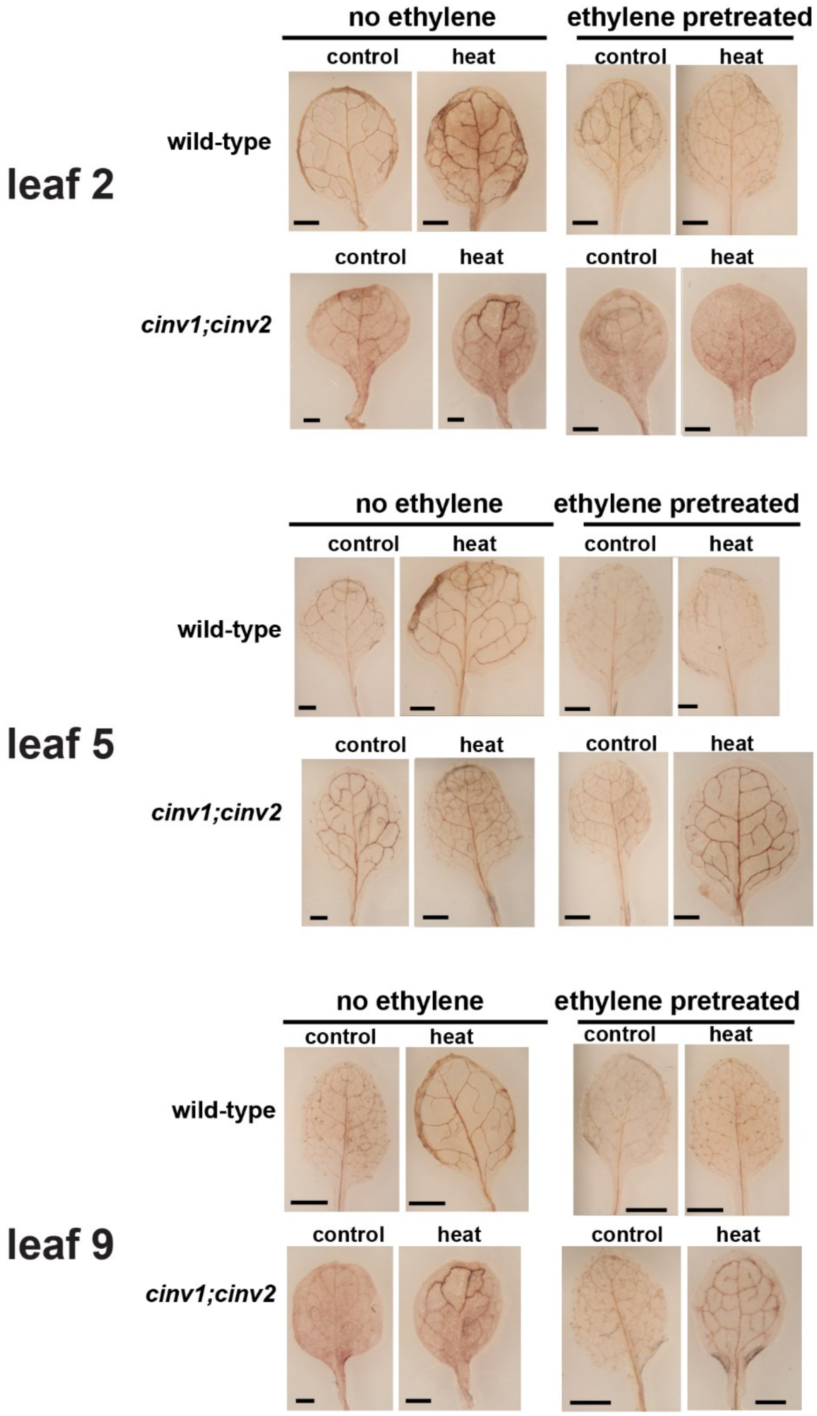

S1 Fig. Images of DAB-stained leaves. Germinating wild-type and *cinv1;cinv2* seeds were exposed to 0.7 ppm ethylene (ethylene pretreated) or ethylene-free air (no ethylene) in darkness for three days. At this time, they were transferred to ethylene-free conditions and grown under a 16 h photoperiod for five days. They were then exposed to either high temperature (45 °C) or control conditions (22 °C) for 30 minutes in darkness. One hour later the samples were stained with DAB to visualize H_2O_2 and images acquired. Representative images are shown. Scale bars = 1 mm.
