# Supplementary material for: CINV1 and CINV2 are required for increased tolerance to diverse stresses after ethylene-pretreatment of germinating seeds
Source: PLoS One. 2025 Jul 15;20(7):e0328236. doi: 10.1371/journal.pone.0328236 (PMC12262900; doi:10.1371/journal.pone.0328236)
Supplement: S2 Fig — (PDF) [file pone.0328236.s002.pdf]

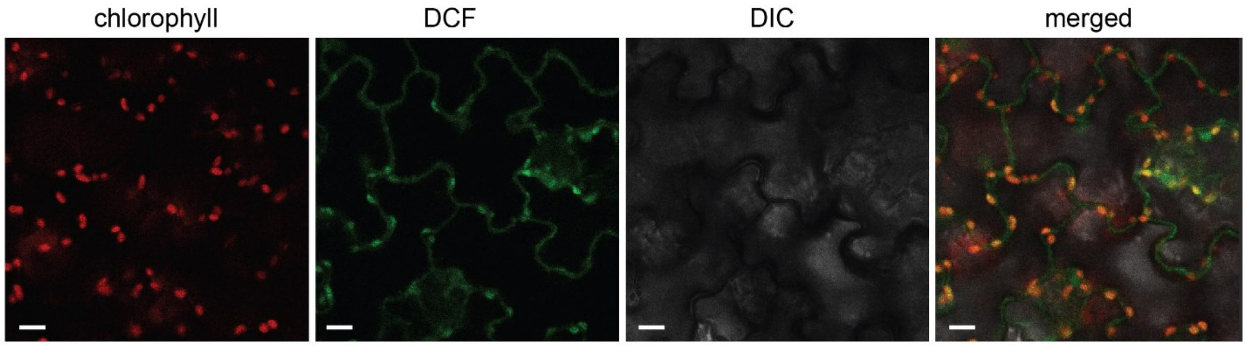

S2 Fig. Close-up of DCF fluorescence in heat-stressed cinv1;cinv2 not pretreated with ethylene. Closeup of one panel from figure 5B of heat-stressed *cinv1;cinv2* showing separate channels for chlorophyll fluorescence, DCF fluorescence, DIC, and merged. Scale bars = 10 µm.
